# A High-Throughput Regeneration and Transformation Platform for Production of Genetically Modified Banana

**DOI:** 10.3389/fpls.2015.01025

**Published:** 2015-11-26

**Authors:** Jaindra N. Tripathi, Richard O. Oduor, Leena Tripathi

**Affiliations:** ^1^Bioscience Centre, International Institute of Tropical AgricultureNairobi, Kenya; ^2^Department of Biochemistry and Biotechnology, Kenyatta UniversityNairobi, Kenya

**Keywords:** banana, *Agrobacterium*-mediated transformation, embryogenic cell suspension, immature male flowers, multiple buds

## Abstract

Banana (*Musa* spp.) is an important staple food as well as cash crop in tropical and subtropical countries. Various bacterial, fungal, and viral diseases and pests such as nematodes are major constraints in its production and are currently destabilizing the banana production in sub-Saharan Africa. Genetic engineering is a complementary option used for incorporating useful traits in banana to bypass the long generation time, polyploidy, and sterility of most of the cultivated varieties. A robust transformation protocol for farmer preferred varieties is crucial for banana genomics and improvement. A robust and reproducible system for genetic transformation of banana using embryogenic cell suspensions (ECS) has been developed in this study. Two different types of explants (immature male flowers and multiple buds) were tested for their ability to develop ECS in several varieties of banana locally grown in Africa. ECS of banana varieties “Cavendish Williams” and “Gros Michel” were developed using multiple buds, whereas ECS of “Sukali Ndiizi” was developed using immature male flowers. Regeneration efficiency of ECS was about 20,000–50,000 plantlets per ml of settled cell volume (SCV) depending on variety. ECS of three different varieties were transformed through *Agrobacterium-*mediated transformation using *gus*A reporter gene and 20–70 independent transgenic events per ml SCV of ECS were regenerated on selective medium. The presence and integration of *gus*A gene in transgenic plants was confirmed by PCR, dot blot, and Southern blot analysis and expression by histochemical GUS assays. The robust transformation platform was successfully used to generate hundreds of transgenic lines with disease resistance. Such a platform will facilitate the transfer of technologies to national agricultural research systems (NARS) in Africa.

## Introduction

Banana and plantain (*Musa* spp.) are the eighth most important staple food and cash crops in tropical and subtropical countries (FAOSTAT, 2013; Tripathi et al., 2014a). They are produced in more than 140 countries and territories across the globe with an annual production of about 144 million tones (FAOSTAT, 2013). The crop is mainly grown by smallholder farmers for food and domestic markets. Uganda is the largest banana producer in Africa with about 10 million tones harvested from over 1.8 million ha (FAOSTAT, 2012). Furthermore, Uganda is known for having the highest consumption rate of 1.6 kg for an individual per day (FAOSTAT, 2001).

Banana production is constrained by various biotic stresses, such as fungal, bacterial, and viral diseases and pests such as weevils and nematodes (Jones, 2000; Tushemereirwe et al., 2004). Currently, banana production in east and central Africa is devastated by the banana *Xanthomonas* wilt (BXW) disease caused by *Xanthomonas campestris* pv. *musacearum*. BXW disease affects the livelihoods of millions of farmers (Tripathi et al., 2009). About 30–60% of yield losses in banana production are also due to abiotic stresses such as drought and soil fertility (Kalyebara et al., 2007). Because banana is an important staple food crop, there is a need to develop varieties for disease resistance and enhanced yield. The improved varieties can be developed through conventional breeding or/and transgenic technologies. However, genetic improvement of banana for disease and pest resistance has not been adequately supported by a successful conventional breeding strategy. The recalcitrant nature of banana in conventional breeding has been attributed to the long generation time, limited genetic variability, absence of disease, or pest resistance in banana germplasm, sterility, and various levels of ploidy (Lorenzen et al., 2010). Due to many restrictions using conventional breeding, the advancement of effective regeneration and genetic transformation provides a substitute for banana improvement. The regeneration system is a critical factor for the production of any genetically modified plant (Hansen and Wright, 1999).

Genetic transformation of banana can be achieved using different methods, such as *Agrobacterium*-mediated transformation, electroporation, and micro-projectile bombardment. However, *Agrobacterium*-mediated transformation is the most preferred method due to its advantages, such as integration of low copy number of transgenes into the host plant genome and transfer of relatively large segments of DNA with minimal rearrangements (Lindsey, 1992; Gelvin, 2003). Several protocols for *Agrobacterium-*mediated transformation are available using different explants such as embryogenic cell suspension (ECS) cultures (Ganapathi et al., 2001; Khanna et al., 2004; Kosky et al., 2010; Tripathi et al., 2012) and apical meristematic tissues for various varieties of banana and plantain (May et al., 1995; Tripathi et al., 2005, 2008). The most commonly used target tissue for transformation is ECS; however, production of such cell suspensions is laborious, time consuming, and extremely variety dependent. Production of ECS and the optimization of a transformation system for each specific variety therefore becomes a prerequisite for genetic improvement. Cell suspensions of various varieties have been developed using basal leaf sheaths and corm section (Novak et al., 1989), highly proliferating multiple meristems (Dheda et al., 1991; Strosse et al., 2006), zygotic embryos (Marroquin et al., 1993), and immature male flowers (Escalant et al., 1994; Cote et al., 1996; Navarro et al., 1997; Becker et al., 2000; Grapin et al., 2000).

The availability of a robust transformation protocol for any variety is a must to produce large numbers of independent transgenic events on a regular basis. We report here a robust and reproducible system for genetic transformation of banana using ECS of “Gros Michel,” “Cavendish Williams,” and “Sukali Ndiizi.” An efficient system for developing ECS using either immature male flowers or highly proliferating multiple buds was established. This is the first comparative study where both immature flowers as well as multiple buds were used to develop embryogenic cells. The amenability of these ECS for transformation and regeneration was investigated to provide a complete system to develop large numbers of transgenic plants with economically useful traits.

## Materials and methods

### Plant materials

Immature male buds of banana varieties “Sukali Ndiizi” (AAB genome), “Cavendish Williams,” “Gros Michel,” (AAA genome), and “Ngombe” (AAA-EAHB) were collected from banana growing fields at Kenya Agricultural and Livestock Research Organization (KALRO), Kenya. Varieties “Cavendish Williams,” “Gros Michel,” and “Mpologoma” (AAA-EAHB) were obtained from the IITA collection as *in vitro* plantlets and used to develop highly proliferating multiple buds.

### Preparation of multiple buds and immature male flowers

The *in vitro* plantlets of varieties “Cavendish Williams,” “Gros Michel,” and “Mpologoma” were micropropagated as described by Tripathi et al. (2012). Small buds produced at the base of the shoot were transferred to multiple bud induction medium (MBI, Supplementary Table 1) and cultured in the dark at 26 ± 2°C. The multiple buds were sub-cultured on MBI medium at 4-week intervals until groups of tiny buds were obtained. About 3–5 monthly subcultures were done to acquire good quality multiple buds.

For immature male flowers, male inflorescences of varieties “Sukali Ndiizi,” “Cavendish Williams,” “Gros Michel,” and “Ngombe” were collected from the field within a month after bunch appearance. The outermost part of the inflorescence was removed and floral apices were surface sterilized with 70% ethanol for 2 min. The floral apices were then washed in sterile distilled water thrice. The floral buds were reduced in size (about 2 cm in length) by removing bracts under sterile conditions.

### Development of embryogenic callus

Multiple buds were isolated on Callus Induction Medium (CIM1, Supplementary Table 1) for initiation of friable embryogenic calli as described by Tripathi et al. (2012). Three hundred explants for each variety were cultured in each experiment. A total of 900 explants were used in three experiments. The cultures were kept in the dark until callus initiated without changing any medium. The cultures were inspected consistently for development of friable embryogenic calli.

For immature male flowers, tiny flowers were isolated under stereomicroscope and cultured on Callus Induction Medium (CIM2). About 6–9 tiny flowers were incubated per 90-mm petri dish, and a total of 300 explants were cultured for callus induction in three experiments for each variety. The cultures were kept in the dark at 26 ± 2°C until callus was obtained without sub-culturing. The cultures were checked biweekly for development of friable embryogenic calli.

### Development of embryogenic cell suspension

Creamish yellow, translucent friable embryogenic calli of each variety were identified under the microscope and transferred into a 25 ml conical flask containing liquid Callus Induction Medium (LCIM1 or LCIM2 depending upon the explants). Initially a 5 ml medium was used in each 25 ml conical flask up to 1 week; gradually the medium was increased to 10 ml in 3 weeks. On the fourth week, fine granular cells were transferred into a new 25 ml conical flask. After 8 weeks of culture, fine cells were transferred into 250 ml conical flasks containing 30–40 ml medium for further proliferation and maturation. These embryogenic cells were washed and replenished with a new medium every 10–14 days (Tripathi et al., 2012).

### Testing of regeneration capacity of ECS of various varieties

The concentration of fast dividing embryogenic cells was adjusted to 3–5% SCV with either LCIM1 or LCIM2. About 1 ml of SCV of the diluted ECS of each variety (“Sukali Ndiizi,” “Cavendish Williams,” and “Gros Michel”) was spread on nylon mesh and cultured on semisolid Embryo Development Medium (EDM, Supplementary Table 1) in 90 mm Petri dish for 1–2 months. The embryos developed on EDM were regenerated into complete plantlets as described by Tripathi et al. (2012). The regeneration efficiency of each variety was calculated as number of plantlets regenerated per ml SCV. The experiments were repeated three times.

### *Agrobacterium* strain and plasmid

The binary vector pCAMBIA2301 containing the *neomycin phosphotransferase* (*npt*II) gene as the selectable marker and β-*glucuronidase* (*gus*A) gene as a reporter was used for all the transformation experiments (Supplementary Figure 1). The culture of *Agrobacterium tumefaciens* strain EHA 105 harboring pCAMBIA2301 was prepared for transformation as described by Tripathi et al. (2012).

### Transformation, selection, and regeneration

Embryogenic cell suspensions of the varieties “Cavendish Williams,” “Gros Michel,” and “Sukali Ndiizi” were transformed through *Agrobacterium*-mediated transformation followed by selection and regeneration of transgenic lines using a modified protocol of Tripathi et al. (2012). The modification was that after co-cultivation of ECS with *Agrobacterium, Agro*-infected embryogenic cells were transferred on EDM without antibiotic selection for 1 week; after that these cells were shifted to selective medium supplemented with 100 mg/l kanamycin. Shoots obtained on selective medium were shifted to proliferation medium (PM, Supplementary Table 1) for multiplication and maintenance. Transformation efficiency was calculated as number of PCR positive transgenic lines regenerated on kanamycin selective medium per ml SCV of ECS of each cultivar. Transformation experiments were repeated three times. The putative transgenic shoots were then transferred to rooting medium (RM, Supplementary Table 1). Rooted plantlets of transgenic and non-transgenic control lines of “Cavendish Williams,” “Gros Michel,” and “Sukali Ndiizi” were transferred to sterile soil in pots as reported by Tripathi et al. (2014a). Plant morphology was compared between transgenic and non-transgenic control plants.

### Histochemical GUS assay

Transient and stable GUS assay was performed with embryogenic cells and with different parts of transgenic plant tissues as described (Jefferson, 1987; Tripathi et al., 2012). Transient expression of *gus*A gene was examined in *Agro*-infected embryogenic cell suspension after 3 days of co-cultivation, while stable expression of the reporter gene was investigated in leaves, petioles, pseudostem, and roots isolated from putative transgenic plants regenerated on selective medium. Non-transformed ECS, leaves, and roots of non-transgenic control plants were also included in the experiment. Photographs were taken using stereomicroscope SMZ1500 attached to a Nikon camera and computer.

### PCR analysis of transgenic plants

Plant genomic DNA was isolated from regenerated putative transgenic fresh leaves using a DNeasy kit (Qiagen, GmbH, Germany). PCR was performed using *gus*A gene specific primers [forward primer 5′- TTTAACTATGCCGGGATCCATCGC -3′ and reverse primer 5′- CCAGTCGAGCATCTCTTCAGCGTA -3′] (Nyaboga et al., 2014). PCR was performed in a total volume of 20 μl, containing 100 ng genomic DNA, 2 μl 10X buffer, 0.5 μl of 10 mM dNTP, 0.5 μl of 10 μM of each primers, and 1 unit of Hot star *Taq* DNA polymerase (Qiagen, Germany). The PCR conditions were: initial denaturation at 95°C for 10 min, 35 cycles of denaturation at 94°C for 15 s, annealing at 62°C for 45 s and extension at 72°C for 50 s followed by final extension at 72°C for 7 min and holding at 4°C. The amplified PCR products were separated by electrophoresis on 0.8% (w/v) agarose gel. Both positive (plasmid DNA) and negative (gDNA of non-transgenic control plant) were included in each experiment.

### Dot blot and southern blot analysis

The integration of *gus*A gene into the genome of banana was analyzed using dot blot and Southern hybridization. Dot blot was performed using gDNA extracted from 24 PCR positive transgenic lines as described by Nyaboga et al. (2014).

For Southern blot analysis, gDNA was isolated from fresh leaves of *in vitro* grown transgenic plants using cetyltrimethylammonium bromide (CTAB) method developed by Gawel and Jarret (1991). About 25 μg of gDNA of transgenic lines and non-transgenic control plant were restricted using enzyme *Hind*III (New England Biolabs, USA). Southern blot analysis was performed as described by Tripathi et al. (2014a).

### Statistical analysis

All the experiments were repeated three times and data were analyzed using the statistical program Minitab 14. The means and standard error presented were for three experiments. One-way analysis of variance (ANOVA) was performed and interaction between means were separated by least significant difference (LSD) at *P* = 0.05.

## Results and discussion

Genetic improvement of bananas is crucial to create new varieties possessing traits of agronomic importance, such as high yield, combined with resistance or tolerance to biotic and abiotic stresses. Other desirable features include better fruit quality, early maturity, short stature, photosynthetic efficiency, strong roots, and uniform fruits (Pillay et al., 2002; Bakry et al., 2009). Conventional plant breeding in banana is challenging due to long generation period, polyploidy, parthenocarpy, low genetic variability, and sterility. Genetic transformation can complement conventional breeding for developing improved varieties of banana. It is also a useful tool for functional genomics (Peraza-Echeverria et al., 2007; Roux et al., 2008; Santos-Rosa et al., 2009). Therefore, it is essential to establish a high throughput regeneration and genetic transformation system, which can be applicable for many farmer-preferred varieties of banana and plantain.

Even though *Agrobacterium tumefaciens*-mediated genetic transformation of banana and plantain has been developed (Ganapathi et al., 2001; Khanna et al., 2004; Tripathi et al., 2012), the transformation efficiency of several farmer-preferred varieties, is still inadequate, and only a limited number of varieties has been genetically modified. Several factors such as variety, age of the explants, *Agrobacterium* strains, plasmid vector, the selectable marker genes, selection agent, and regeneration conditions affect the efficiency of genetic transformation (Hiei et al., 1997; Cheng et al., 2004). Most of the transformation protocols are variety dependent and it is difficult to overcome this by using only highly virulent strains of *Agrobacterium* (Hansen et al., 1994; Liu and Nester, 2006; Nyaboga et al., 2014) or optimizing plant regeneration conditions (Lee et al., 2002; Zuo et al., 2002). Therefore, there is a need to optimize the protocol for each economically important variety.

### Development of ECS for various banana varieties

**Figure 1:**
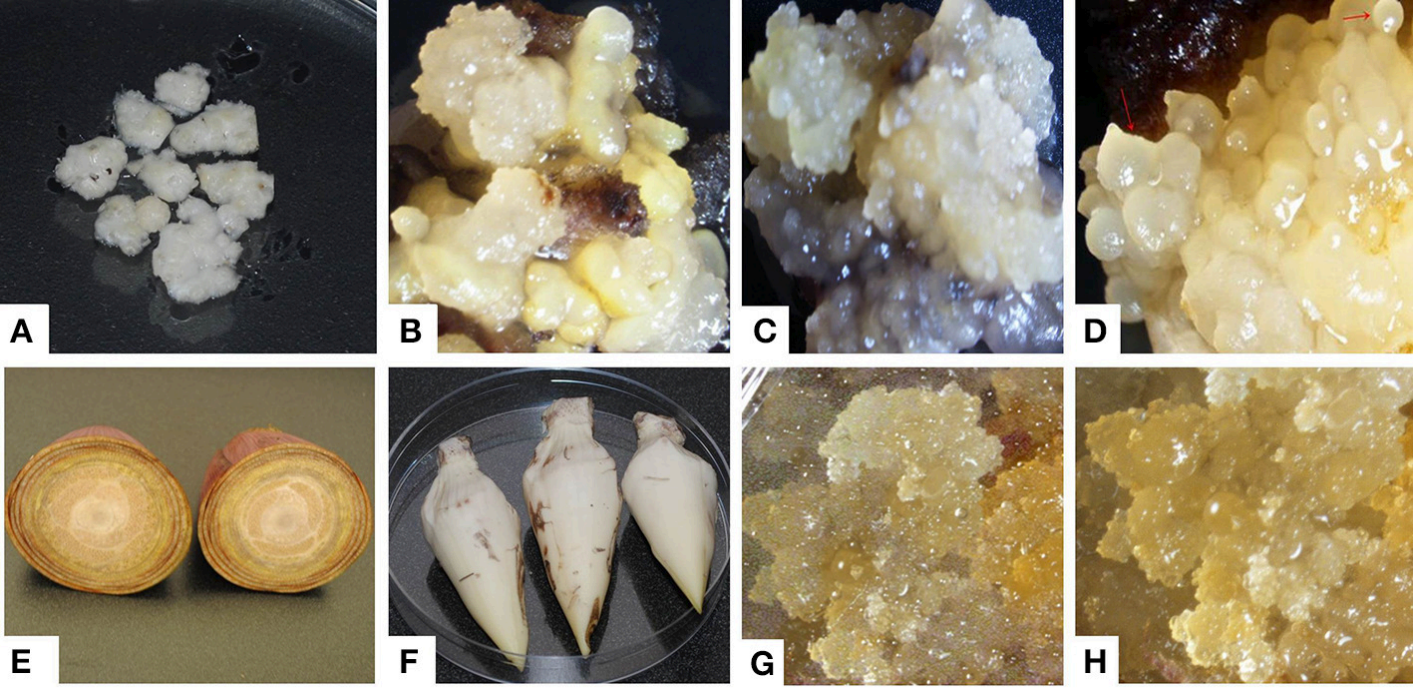
**Induction of embryogenic calli using multiple buds and immature male flowers of various banana varieties**. **(A)** Multiple buds, **(B)** embryogenic calli of “Cavendish Williams,” **(C)** embryogenic calli of “Gros Michel,” **(D)** embryogenic calli of “Mpologoma,” **(E,F)** banana inflorescences, **(G)** embryogenic calli of “Sukali Ndiizi” and **(H)** embryogenic calli of “Ngombe.”

**Figure 2:**
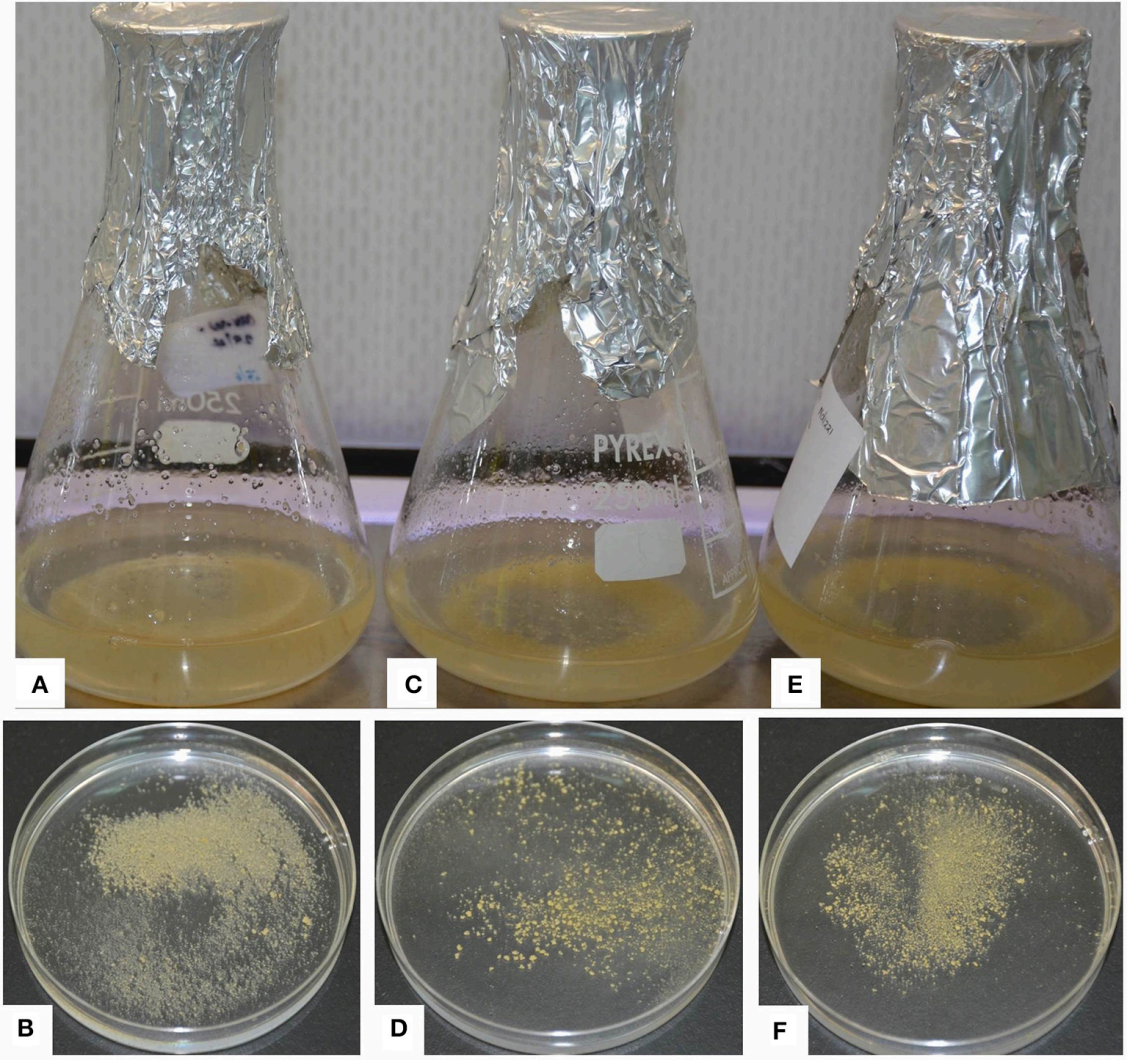
**Embryogenic cell suspensions (ECS) of various varieties of banana. (A)** ECS of “Cavendish Williams” in liquid, **(B)** ECS of “Cavendish Williams” on plate, **(C)** ECS of “Gros Michel” in liquid, **(D)** ECS of “Gros Michel” on plate, **(E)** ECS of “Sukali Ndiizi” in liquid, **(F**) ECS of “Sukali Ndiizi” on plate.

ECS are the best explant for genetic transformation of banana because chimerism can be avoided in this system as transgenic events are obtained from a single cell (Roux, 2004). Immature male flowers from cultivars “Cavendish Williams,” “Gros Michel,” “Sukali Ndiizi,” “Ngombe” and multiple buds from cultivars “Cavendish Williams,” “Gros Michel,” and “Mpologoma” were tested as explants to obtain friable embryogenic callus and ECS (Figure 1 and Table 1). Yellow creamish friable calli were developed using immature male flowers from banana cultivars “Cavendish Williams,” “Gros Michel,” “Ngombe,” and “Sukali Ndiizi” in 4–6 months (Figure 1). These friable calli were transferred in LCIM2 for development of ECS. Yellowish and creamish granular embryogenic cells were observed after 1–2 months in “Sukali Ndiizi” (Figure 2). These fine cells increased and uniform cell suspension was obtained in 3–4 months following initiation (Figure 2). However, all calli for “Cavendish Williams,” “Gros Michel,” and “Ngombe” turned black after transferring to liquid medium and no embryogenic cells were observed. This could be due to the quality of calli, which was not good for the production of embryogenic cells.

**Table 1:** **Summary of development of embryogenic cell suspension (ECS) and transformation for various banana varieties**.

**Variety**	**Explant**	**No. of explants cultured**	**No. of explants responded for callus induction**	**No. of calli transferred to liquid callus induction medium**	**No. of calli developed ECS**	**ECS established**	**Transformation established**
Cavendish Williams	Multiple buds	900	850 (94%)	60	30 (50%)	Yes	Yes
Cavendish Williams	Immature male flowers	300	36 (12%)	36	All calli turned brown and black	No	No
Gros Michel	Multiple buds	900	630 (70%)	50	22 (44%)	Yes	Yes
Gros Michel	Immature male flowers	300	30 (10%)	30	All calli turned brown and black	No	No
Mpologoma	Multiple buds	900	432 (48%)	50	All calli turned brown and black	No	No
Ngombe	Immature male flowers	300	22 (7%)	10	All calli turned brown and black	No	No
Sukali Ndiizi	Immature male flowers	300	45 (15%)	45	3 (6.6%)	Yes	Yes

Friable calli were developed using multiple buds of “Cavendish Williams,” “Gros Michel,” and “Mpologoma” after 3–5 months of culture of explants in the dark (Figure 1). Fine embryogenic cells were obtained from creamish yellowish calli in about 1–2 months after transferring them to LCIM1. Embroygenic cells of “Cavendish Williams” and “Gros Michel” were multiplied and the uniform cell suspensions were established in 3–4 months after initiation (Figure 2). However, the calli of “Mpologoma” turned brownish black and did not produce any embryogenic cells.

The ECS developed for different cultivars using either of the explants were maintained for 1–1.5 years by subculturing every 10–14 days. The quantity of embryogenic cells was doubled every 2 weeks, but the regeneration capacity of ECS was observed to decrease with time. In this study, we noticed that both the explants were amenable for development of embryogenic cells depending upon the cultivar (Table 1). However, multiple buds are preferred explants as they can be easily produced using tissue culture plantlets under laboratory conditions. To get immature male flowers, researchers need to have access to field-grown plants, which is sometimes not possible. Previous reports demonstrated the development of embryogenic cell suspension of “Cavendish Williams” using immature male flowers (Xu et al., 2005; Ghosh et al., 2009; Youssef et al., 2010; Chong-Pe'rez et al., 2012). However, we could not obtain ECS of “Cavendish Williams” from immature male flowers, but we successfully obtained ECS from multiple buds. In this study, we compared two types of explants, multiple buds and immature male flowers, for development of ECS with various cultivars. ECS were obtained from both multiple buds and immature male flowers depending upon cultivar (Table 1).

### Regeneration capacity of ECS of different varieties

**Figure 3:**
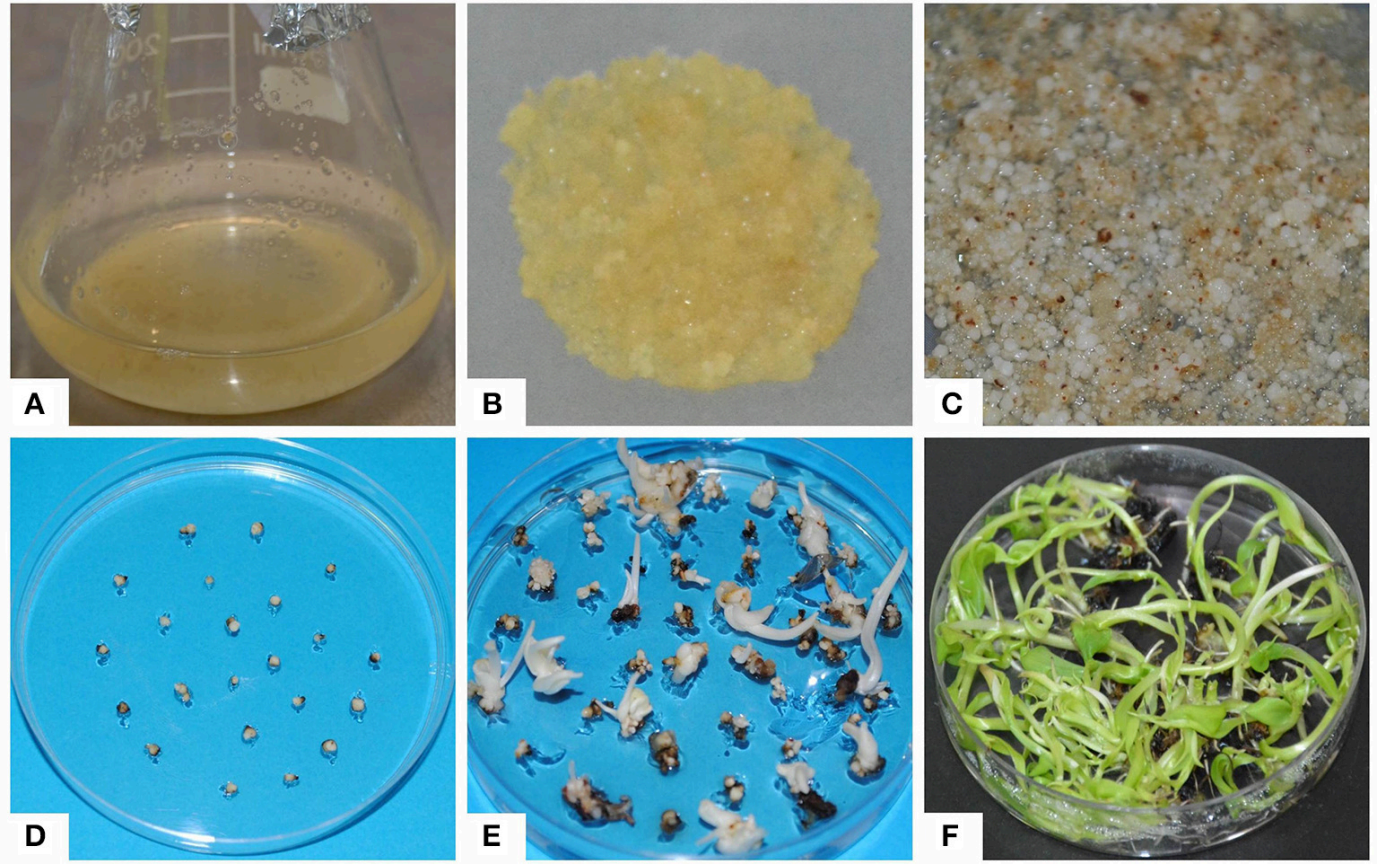
**Regeneration of embryogenic cell suspensions**. **(A)** Embryogenic cell suspension, **(B)** embryogenic cells plated on nylon mesh cultured on embryo development medium, **(C)** regenerating white embryos on embryo development medium, **(D)** embryos on embryo maturation medium, **(E)** embryos on germination medium, **(F)** regenerated shoots on proliferation medium.

**Figure 4:**
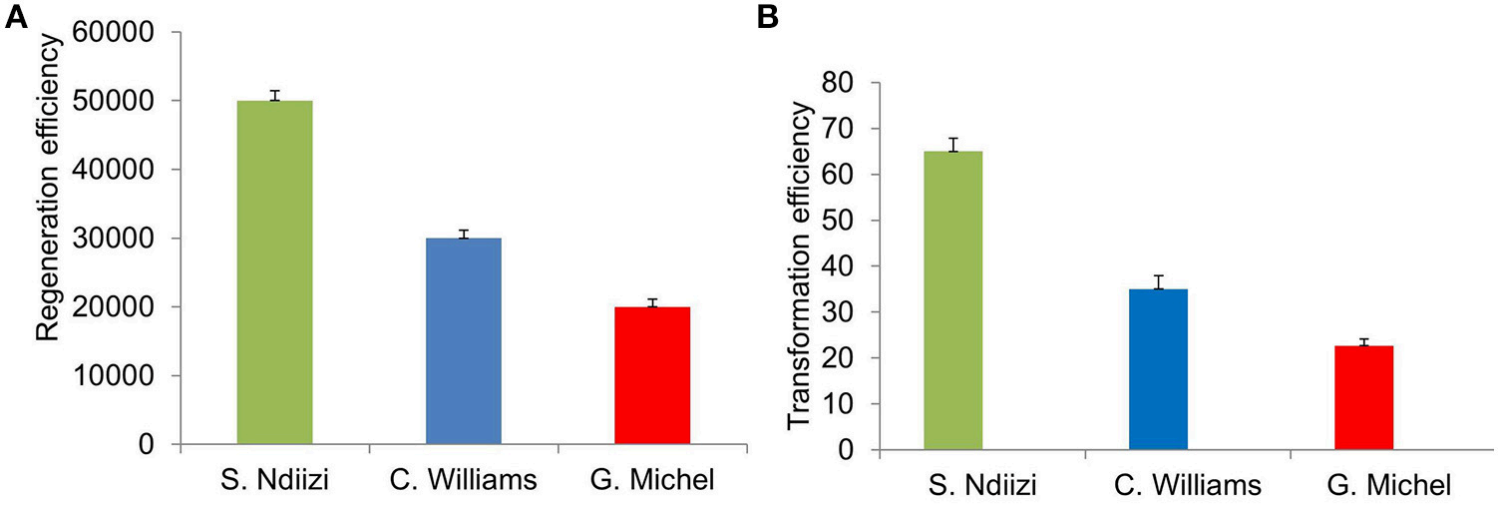
**Graphs showing regeneration and transformation efficiency of various banana varieties using embryogenic cell suspension (ECS). (A)** Regeneration efficiency as number of plantlets regenerated from 1 ml of settled cell volume of ECS and **(B)** transformation efficiency as number of transgenic lines generated from 1 ml of settled cell volume of ECS transformed. Values were presented mean ± S.E.

Three-month old ECS of “Cavendish Williams,” “Gros Michel,” and “Sukali Ndiizi” were tested for regeneration and their regeneration efficiencies were compared (Figures 3, 4). White globular embryos appeared in 1–2 months on EDM, which were then transferred to Embryo Maturation Medium (EMM, Supplementary Table 1) for 1 month. The mature white embryos were cultured on Embryo Germination Medium (EGM, Supplementary Table 1), where they started germinating in 1–2 months and produced whitish shoots which were subsequently transferred onto proliferation medium for shoot development (Figure 3). We observed that once cells suspensions were established, regeneration of complete plantlets were similar irrespective of explants from which cell suspensions were originated.

The regenerated shoots were transferred to light with photoperiod of 16 h where they turned green and subsequently transferred onto rooting medium for 2–4 weeks. About 99% of well-rooted plants were successfully hardened and established in pots in the glasshouse. The plants exhibited identical morphology compared with other plants developed using suckers.

Approximately 20,000–50,000 plantlets were developed from 1 ml SCV of ECS (Figure 4A). Maximum numbers of plantlets (about 50,000 plantlets per ml SCV) were regenerated from ECS of “Sukali Ndiizi,” whereas “Gros Michel” was found to be least responsive, developing about 20,000 plantlets per ml SCV. Approximately 30,000–40,000 plantlets per ml SCV were regenerated for “Cavendish Williams.” It was noted that regeneration efficiency is cultivar dependent, which was also observed in previous reports. Regeneration of 25,000–30,000 plants per 1 ml of SCV was reported for plantain cultivars (Strosse et al., 2006; Tripathi et al., 2012).

In this study, we obtained complete plantlets from ECS in 13–15 months for different banana varieties, similar to our previous report (Tripathi et al., 2012). We have observed somaclonal variations such as retarded growth, and thinner and variegated leaves in about 3–5% regenerated plants in the glasshouse. To minimize somaclonal variation, we use ECS for transformation and regeneration only for 1–1.5 years after their establishment. Production of embryogenic cell suspension cultures and regeneration is time intensive and variety dependent. It has been reported previously that regeneration of whole plantlets takes 14–42 months for banana and 18–27 months for plantain starting from callus induction (Strosse et al., 2006).

### Transformation, selection, and regeneration of transgenic plant

**Figure 5:**
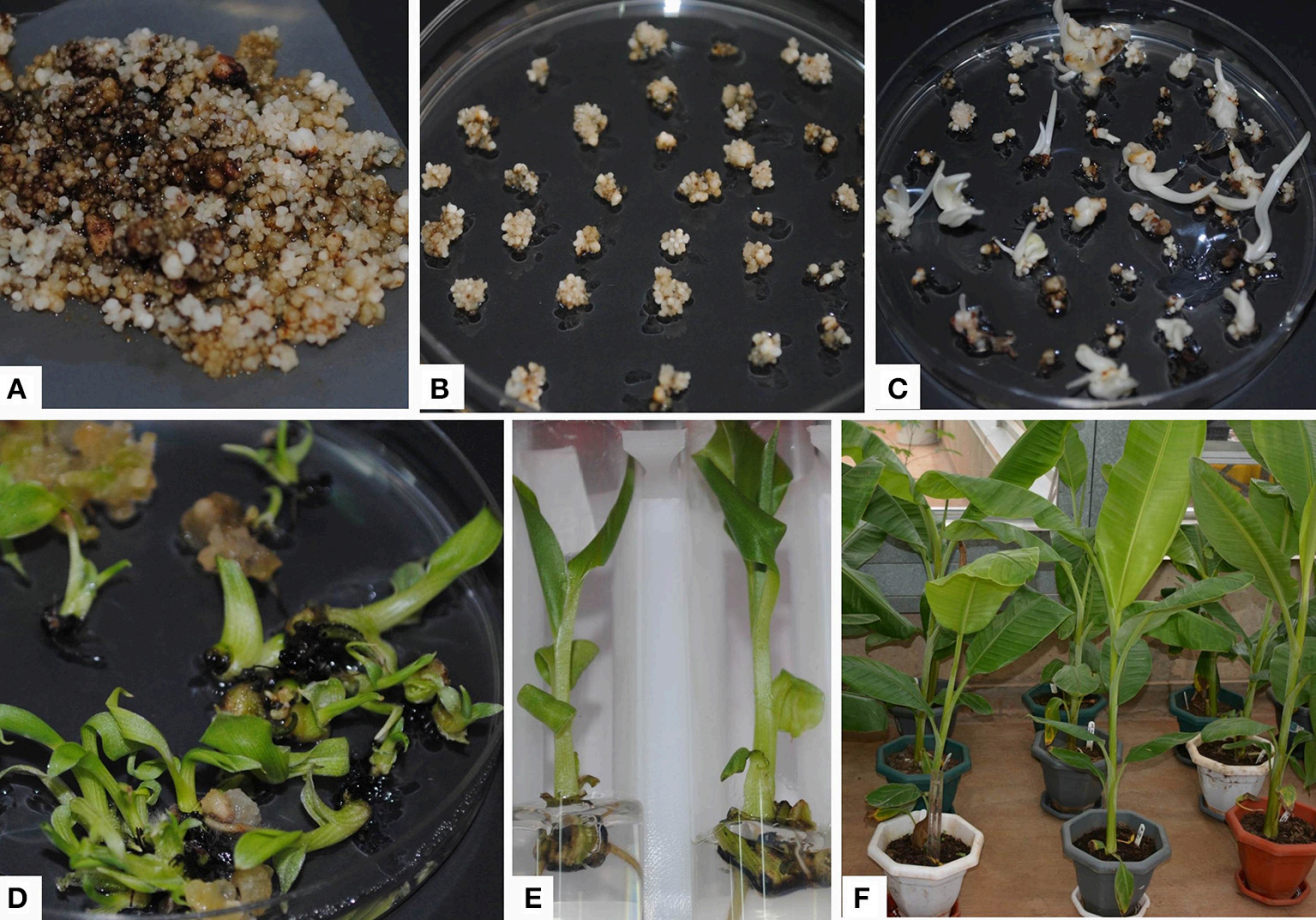
*****Agrobacterium***-mediated transformations of embryogenic cell suspensions. (A)** *Agro-*infected cells proliferating on selection medium, **(B)** embryos on embryo development medium, **(C)** embryos on embryo maturation medium, **(D)** shoot germination on embryo germination medium, **(E)** complete shoots in proliferation medium, **(F)** potted transgenic plants in glasshouse.

ECS of three varieties “Gros Michel,” “Cavendish Williams,” and “Sukali Ndiizi” were transformed with *Agrobacterium tumefaciens* strain EHA 105 containing the pCAMBIA2301 plasmid (Supplementary Figure 1). *Agro*-infected cells multiplied and proliferated on EDM supplemented with kanamycin, whereas the non-transformed control cells turned black. Creamish white colored transformed embryos developed on selective medium in 1–2 months (Figure 5A). These embryos matured in 1 month after culturing them on EMM (Figure 5B) and later on the matured embryos were transferred to EGM (Figure 5C). The germination of putative transgenic plants started 1–2 months after transferring the mature embryos on EGM. The fully developed shoots with few leaves were transferred on proliferation medium (PM) and cultured in a light and dark 16/8 h cycle (Figures 5D–E). The shoots were proliferated and individual shoots were cultured on rooting medium without selection. Roots were developed in 2–4 weeks in all the shoots. Proliferation and elongation of shoots for “Gros Michel” was found to be challenging. Shoots cultured on proliferation medium turned into nodular structures instead of multiplying and elongating. To rectify this challenge, the shoots germinated on EGM were transferred into hormone-free medium for 1 month and then transferred to proliferation medium. This modification provided complete shoots from “Gros Michel.” Kanamycin was noted to have an inhibitory effect on regeneration of transformed shoots in previous studies (Yao et al., 1995; Tripathi et al., 2012), thus after regeneration of complete shoots antibiotic was withdrawn from the proliferation and rooting medium.

Approximately 20–70 transgenic lines per ml SCV were generated depending on the banana variety used in about 7–9 months after *Agro*-inoculation of embryogenic cells. Maximum number of transgenic lines (60–70) was obtained from ECS of “Sukali Ndiizi” and minimum number of lines (20–30) was obtained for “Gros Michel.” About 30–40 lines were developed from 1 ml SCV of embryogenic cells of “Cavendish Williams” (Figure 4B). The transformation efficiency was well-correlated to regeneration efficiency of embryogenic cells of various varieties. Higher regeneration efficiency can provide more independent transgenic shoots. Regeneration efficiency of transgenic lines depends on quality and age of embryogenic cells used for transformation. Newly developed fine cells provide more transgenic plants. Bigger clumps of cells are not good for transformation as they just turned brown and black. The transformation efficiencies obtained in this study were similar to previous reports. About 25–65 plants per 50 mg of SCV of embryogenic suspension cells were reported for variety “Cavendish” and “Lady Finger” (Khanna et al., 2004; Ghosh et al., 2009). Similarly, Ganapathi et al. (2001) reported approximately 40 transgenic plants per 0.5 ml packed cell volume of ECS of variety “Rasthali” (AAB). However, Chong-Pe'rez et al. (2012) recently reported more than 600 independent transgenic lines from 50 mg of settled cells of Dwarf Cavendish.

Fully developed rooted transgenic plants were transferred to pots in the glasshouse and about 96–100% plants were successfully established (Figure 5F). Morphologically all the transgenic plants were similar to control non-transgenic control plants. Transformation experiments were repeated three times using several lines of cells, confirming the reproducibility of the developed transformation system.

**Figure 6:**
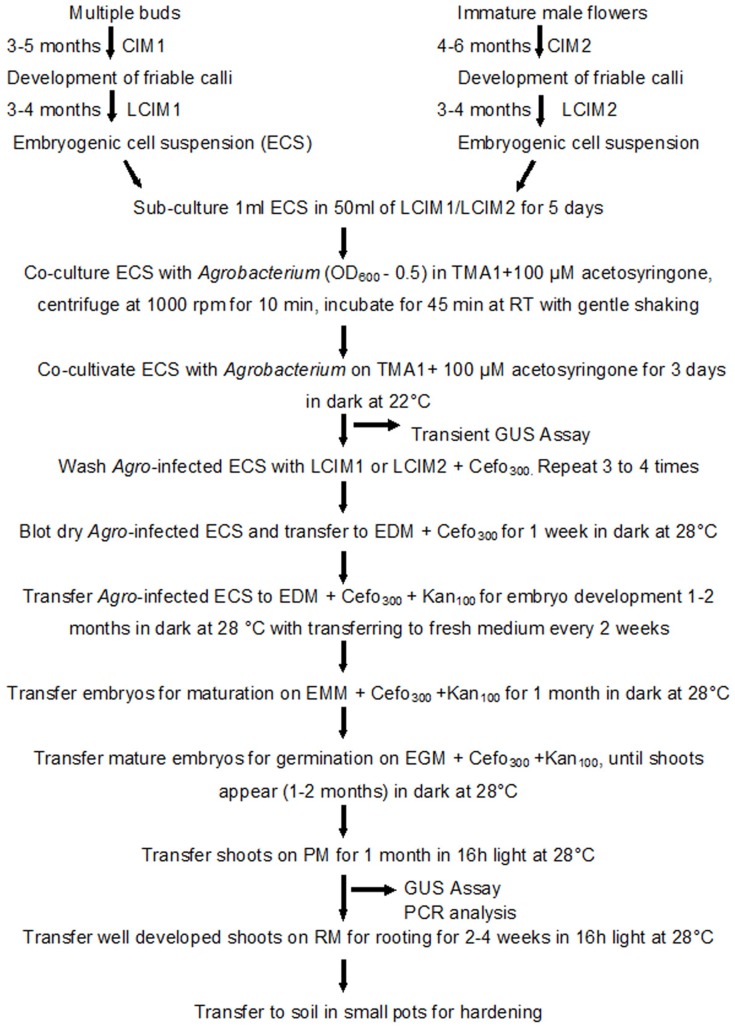
**Schematic diagram showing various steps of genetic transformation of banana using embryogenic cell suspensions (ECS) derived from multiple buds or immature male flowers**. CIM, callus induction medium; LCIM, liquid callus induction medium; TMA1, *Agrobacterium* resuspension medium; EDM, embryo development medium; EMM, embryo maturation medium; EGM, embryo germination medium; RM, rooting medium; Cefo_300_, 300 mg/l cefotaxime; Kan_100_, 100 mg/l Kanamycin.

In this study we used the same protocol to transform ECS and regenerate complete plants, regardless of explants from where ECS was derived. The detailed protocol for transformation and regeneration is presented in Figure 6.

### Histochemical GUS assay

**Figure 7:**
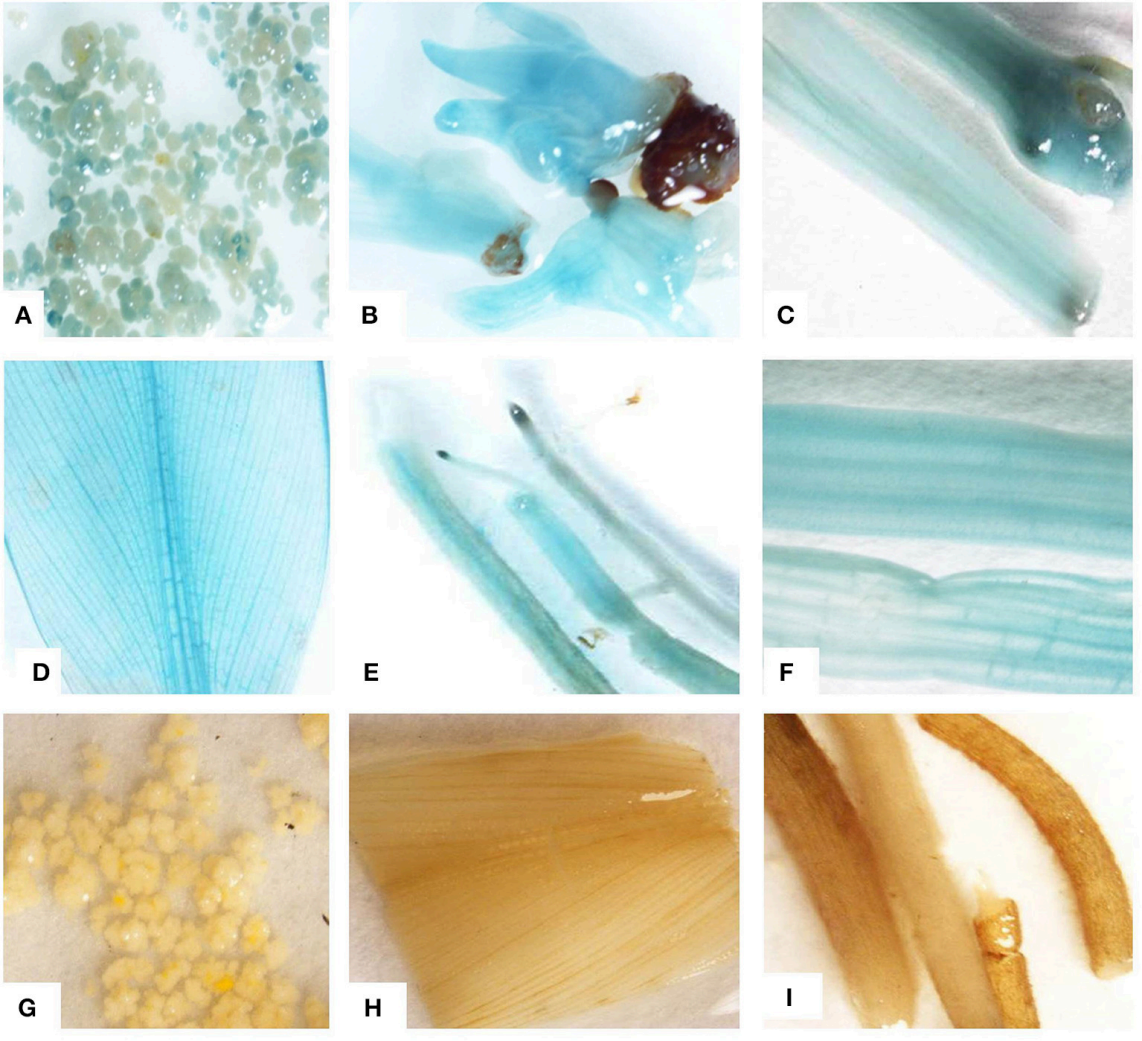
**Histochemical GUS assay**. **(A)** Transient expression of *gus*A gene in embryogenic cells after 72 h of *Agro-*infection, **(B)** stable expression of *gus*A gene in regenerating transgenic embryos, **(C)** pseudostem, **(D)** leaf, **(E)** roots, and **(F)** leaf petioles from transgenic plant, **(G)** non-transformed embryogenic cells, **(H)** leaf, and **(I)** roots from non-transgenic control plant. All the photographs were taken by Nikon SMZ 1500 microscopic camera attached with computer.

GUS assay of *Agro*-infected embryogenic cells showed blue coloration confirming transient expression of the reporter *gus*A gene after 3 days of co-cultivation (Figure 7A). A blue coloration was seen in germinated embryos, leaves, petioles, pseudostems, and roots isolated from transgenic plants regenerated on kanamycin selection medium, confirming stable expression of *gus*A reporter gene (Figures 7B–F). The blue coloration in various tissues of transgenic plants demonstrated uniform transformation. No blue coloration was observed in control non-transformed embryogenic cells (Figure 7G), leaves, and roots of non-transgenic control plants (Figures 7H,I).

### Molecular characterization of transgenic lines

**Figure 8:**
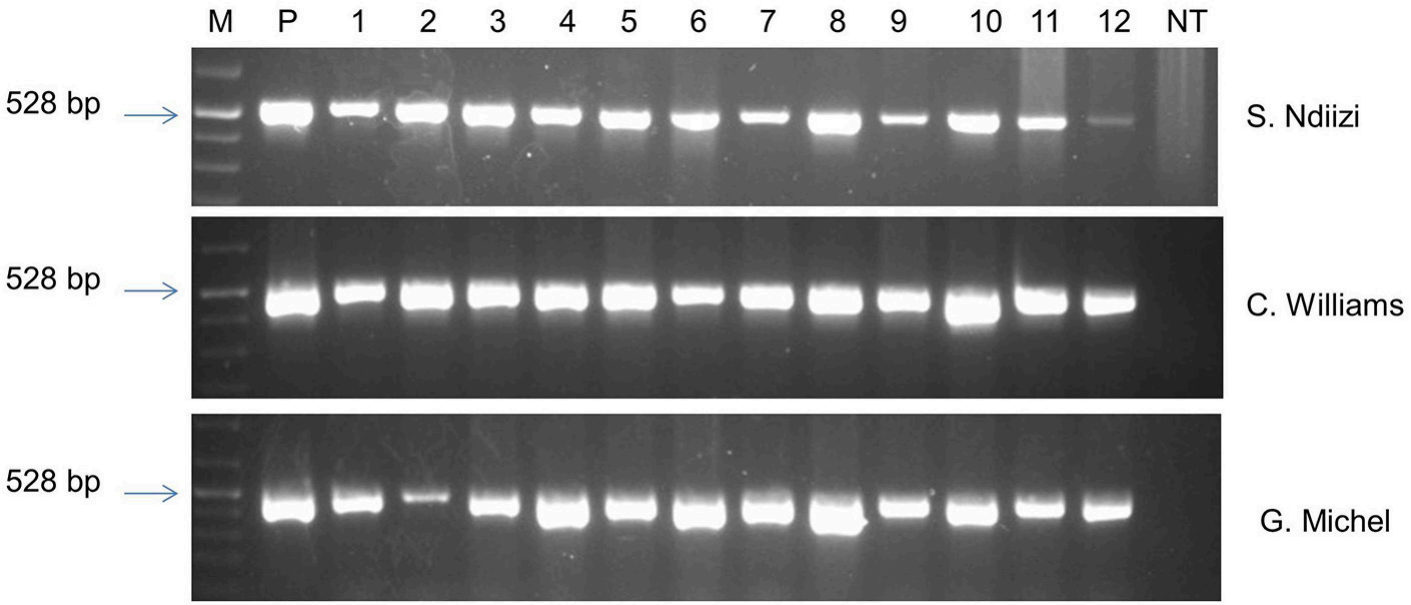
**PCR analysis of genomic DNA of putative transgenic and non-transgenic control plants of various banana varieties using ***gusA*** specific primers**. M, marker; P, plasmid DNA; NT, non-transgenic control plant.

The presence of the transgene in randomly selected transgenic lines of “Sukali Ndiizi,” “Cavendish Williams,” and “Gros Michel” was confirmed by PCR analysis using *gus*A specific primers. The amplicon of expected size of 528 bp was obtained in all the transgenic lines tested validating the presence of *gus*A reporter gene in the genome of transgenic lines (Figure 8). No amplified PCR product was detected in non-transgenic control plants.

**Figure 9:**
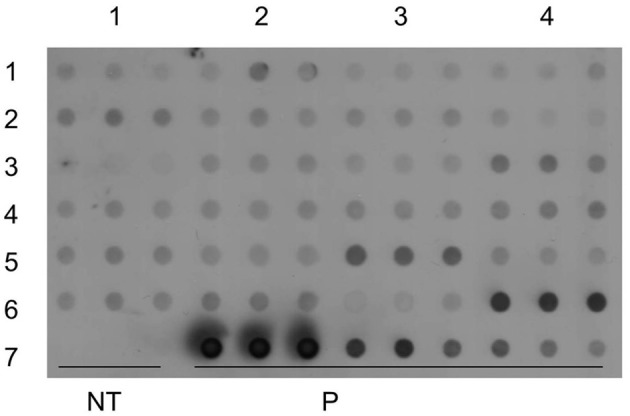
**Molecular analysis of transgenic lines to confirm integration of transgene**. Dot blot analysis of transgenic lines in triplicates, pCAMBIA2301 as a positive control in triplet (10, 5, 1 ng) and control non-transgenic plant.

Dot and Southern blot analysis were performed with genomic DNA isolated from PCR positive transgenic lines. Twenty four transgenic lines along with the positive control and non-transgenic control were tested in three replicates by dot blot analysis. All the transgenic lines tested showed a positive signal except for the control non-transgenic control plant (Figure 9). Dot blot is a rapid technique, which can be used for quick testing of large numbers of transgenic lines for transgene integration.

The gDNA of randomly selected transgenic and non-transgenic control plants were restricted with *Hind*III enzyme. Southern blot analysis was performed and to confirm the transgene integration. *Hind*III has a single restriction site in the pCAMBIA2301 construct used for plant transformation. All 14 independent transgenic lines tested showed positive bands; however, no band was observed for the control non-transgenic control plant (Supplementary Figure 2). The copy number of integrated *gus*A gene was 1–4 in all the tested transgenic lines.

### Testing of robustness of the regeneration and genetic transformation protocol

**Figure 10:**
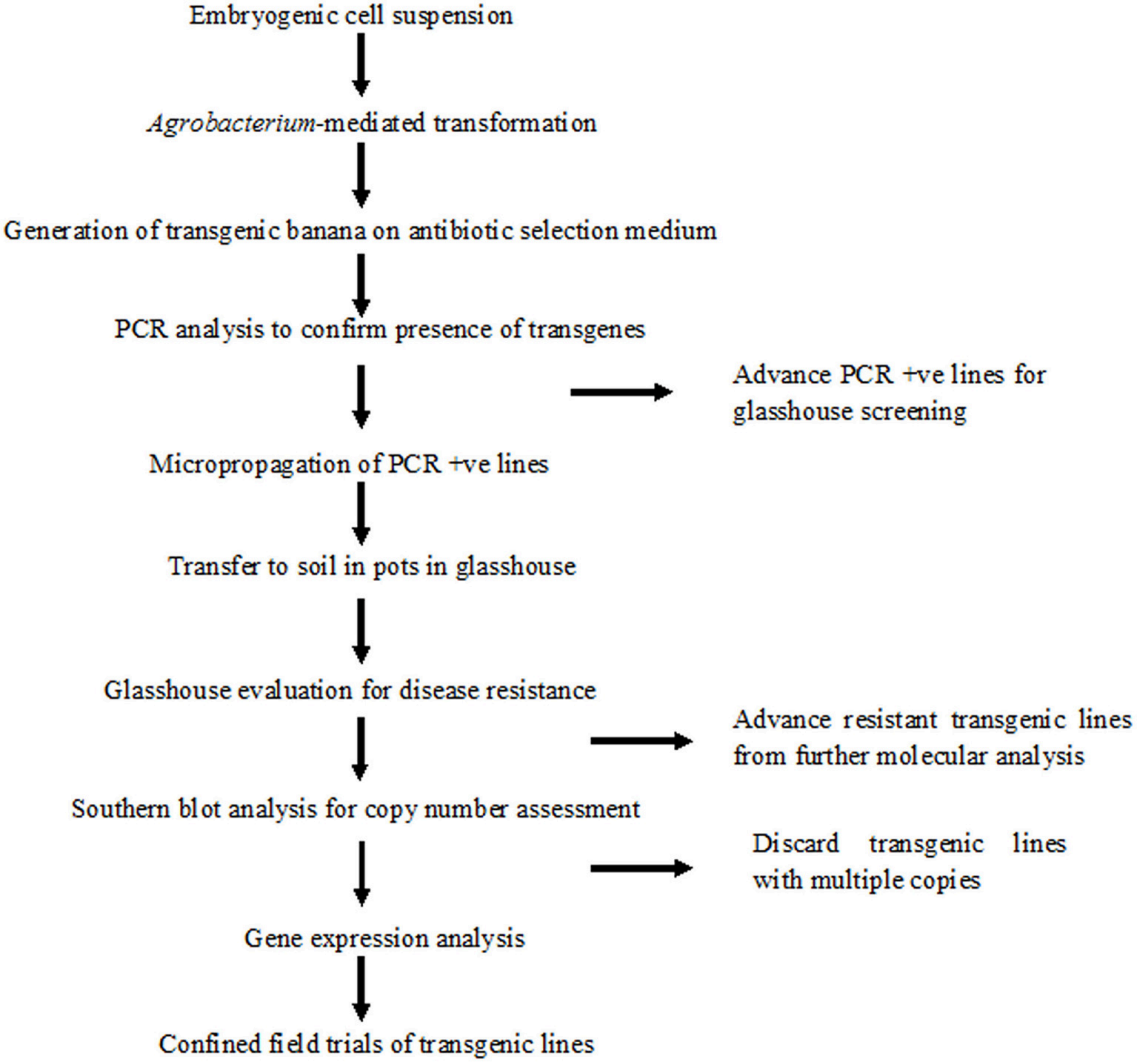
**Schematic representation of production of banana transgenic lines, molecular analysis and evaluation for disease resistance**.

A robust high throughput regeneration and genetic transformation system is required for rapid testing of large numbers of gene constructs in any crop. The main focus of research in our laboratory is to control the deadly banana *Xanthomonas* wilt (BXW) disease, which is the most important biotic constraint to banana production in east and central Africa (Tripathi et al., 2009, 2014b). In the absence of resistant banana germplasm and considering the difficulties associated with conventional breeding, we are applying transgenic technologies to develop BXW resistant bananas using farmer-preferred cultivars. This necessitates the development of a robust regeneration and transformation protocol capable of producing large numbers of independent transgenic lines on a routine basis. The regeneration and transformation platform established in this study has opened the door for genetic advancement of banana by inserting desirable traits such as disease and pest resistance. To validate the reproducibility of this system, various *Agrobacterium*-mediated transformation experiments were performed to develop BXW resistant banana using different gene constructs. In total, 867 transgenic lines were generated from ECS of three cultivars (“Cavendish Williams,” “Gros Michel,” and “Sukali Ndiizi”) with various gene constructs (Table 2). These transgenic lines were tested by PCR analysis using primers specific to target gene and selection marker gene to confirm the presence of all the transgenes. The amplicons of expected sizes provide strong evidence of a complete T-DNA insertion. PCR positive transgenic lines were further evaluated for BXW disease resistance under glasshouse conditions. As hundreds of transgenic lines are produced for testing the efficacy of each gene construct for disease resistance, only lines showing enhanced disease resistance are advanced further for detailed molecular analysis such as Southern blotting, RT-PCR, qRT-PCR, and northern blotting. The transgenic lines showing the presence of transgene/s, enhanced disease resistance, low copy integration, and expression of the transgene were selected for the confined field trial. The various steps of production of transgenic banana, molecular analysis, and evaluation for disease resistance are summarized in Figure 10. Currently, we are also developing large numbers of transgenic banana plantlets using gene constructs with other traits such as virus and fungus resistance for glasshouse screening and confined field trials.

**Table 2:** **Transgenic plants generated using various gene constructs and embryogenic cell suspensions of “Cavendish Williams,” “Gros Michel,” and “Sukali Ndiizi,” in order to develop banana ***Xanthomonas*** wilt resistant transgenic banana**.

**Variety**	**Gene construct**	No. of experiments performed^*^	**No. of transgenic plants generated**	**No. of PCR positive lines**	**No. of transgenic plants tested in glasshouse**
Cavendish Williams	pBI-35S:*Hrap*	2	51	51	35
Cavendish Williams	pBI-35S:*Pflp*	2	75	75	49
Cavendish Williams	pBI-Ubi:*Pflp*::35S:*Hrap*	3	91	91	91
Cavendish Williams	pBI-35S:*Pflp*::35S:*Hrap*	1	47	47	20
Gros Michel	pBI-35S:*Hrap*	4	80	80	20
Gros Michel	pBI-35S:*Pflp*	2	36	36	28
Gros Michel	pBI-Ubi:*Pflp*::35S:*Hrap*	2	50	50	20
Gros Michel	pBI-35S:*Pflp*::35S:*Hrap*	4	100	100	98
Gros Michel	pBI-35S:*EsPflp*	4	100	100	51
Sukali Ndiizi	pBI-35S:*EsPflp*	2	100	100	18
Sukali Ndiizi	pCAMBIA-Ubi:*NH1*	2	112	112	20
Gros Michel	pCAMBIA-Ubi:*NH1*	1	25	25	–

* *1 ml SCV of embryogenic cell suspension was used in each experiment*.

## Conclusion

In conclusion, we developed a high-throughput technique for *Agrobacterium*-mediated transformation and regeneration of economically significant banana varieties grown in east African countries and globally. The ECS of three banana varieties—“Cavendish Williams,” “Gros Michel,” and “Sukali Ndiizi”—which are highly prone to fungal as well as bacterial diseases, were successfully transformed using *Agrobacterium*-mediated system. Transformed ECS have the potential to generate transgenic plants with high efficiency, therefore, only a few milliliters of cells can provide a sufficient number of transformed plants with the desired traits. This high-throughput protocol can be used for functional genomics and improvement of banana varieties. Using this protocol, hundreds of transgenic banana lines with bacterial wilt resistance were generated (Table 2). This study showed potential for genetic manipulation of banana varieties for developing resistance to biotic and abiotic stresses. It is possible that with further optimization, this protocol can be applicable to other varieties of banana in future. This type of high-throughput system also serves as an important platform for transferring technologies to NARS in sub-Saharan Africa on banana improvement.

### Conflict of interest statement

The authors declare that the research was conducted in the absence of any commercial or financial relationships that could be construed as a potential conflict of interest.
