# Supplementary material for: A High-Throughput Regeneration and Transformation Platform for Production of Genetically Modified Banana
Source: Front Plant Sci. 2015 Nov 26;6:1025. doi: 10.3389/fpls.2015.01025 (PMC4659906; doi:10.3389/fpls.2015.01025)
Supplement: Supplementary file 1 [file DataSheet1.DOCX]

**Supplementary Materials**

**Supplementary Table 1:** Composition of various medium used in the banana transformation experiments.

| **Medium** | **Composition** |
| --- | --- |
| Proliferation medium (PM) | MS^1^ salts and vitamins, 100 mg/l myo-inositol, 30 g/l sucrose ,100 mg/l ascorbic acid , 2.5 mg/l BAP and 3 g/l gelrite , pH 5.8 |
| Multiple bud induction medium (MBI) | MS^1^ salts and vitamins, 100 mg/l myo-inositol ,30 g/l sucrose ,100 mg/l ascorbic acid , 24 mg/l BAP and 3 g/l gelrite , pH 5.8 |
| Callus induction medium for multiple buds (CIM1) | MS^1^ salts and vitamins, 100 mg/l myo-inositol , 30g/l sucrose , 10 mg/l ascorbic acid , 1 mg/l 2,4-D , 0.2 mg/l zeatin and 3 g/l gelrite , pH 5.8 |
| Callus induction medium for immature flowers (CIM2) | MS^1^ salts and vitamins, 1 mg/l biotin , 1 mg/l IAA , 4 mg/l 2,4-D , 1 mg/l NAA , 30g/l sucrose, 3g/l gelrite , pH 5.8 |
| Embryo development medium (EDM) | SH^2^ salts, MS vitamins, 100 mg/l glutamine , 100mg/lmalt extract, 230 mg/l proline, 45 g/l sucrose , 10 g/l lactose, 0.05 mg/l zeatin , 0.1 mg/l kinetin , 0.2 mg/l NAA , 0.2 mg/l 2iP , 3 g/l gelrite , pH 5.8 |
| Embryo maturation medium (EMM) | MS^1^ salts and vitamins, 100 mg/l myo-inositol, 30 g/l sucrose , 100 mg/l ascorbic acid and 3 g/l gelrite , pH 5.8 |
| Germination medium (GM) | MS salts, Morel^3^ vitamins, 30 g/l sucrose, 2 mg/l IAA, 0.5 mg/l BAP and 3 g/l gelrite, pH 5.8 |
| Rooting medium (RM) | MS^1^ salts and vitamins, 100 mg/l myo-inositol , 30g/l sucrose , 10mg/l ascorbic acid , 1 mg/l IBA and 3 g/l gelrite, pH 5.8 |
| Yeast extract broth (YEB) | Yeast extract (0.1%), beef extract (0.5%), peptone (0.5%), sucrose (0.5%) and MgSO4 (0.04%) |
| TMA1 | MS^1^ salts and vitamins, 1 mg/l biotin , 100 mg/l malt extract, 100 mg/l glutamine , 230 mg/l proline , 40 mg/l ascorbic acid , 5 g/l PVP10 , 200 mg/l cysteine , 1 mg/l IAA , 1 mg/l NAA , 4 mg/l 2,4-D , 85.5 g/l sucrose , 100µM acetosyringone, pH 5.3 |

^1^Murashige and Skoog, 1962; ^2^Schenk and Hildebrandt, 1972; ^3^Morel and Wetmore,1951.

Abbreviations: BAP - 6-benzylaminopurine; 2,4-D - 2,4-dichlorophenoxyacetic acid; IAA - indole-3-acetic acid; IBA - indole-3-butyric acid; 2iP - isopentenyl adenine; NAA- naphthaleneacetic acid; PVP - polyvinylpyrrolidone

**Supplementary Figure 1:** Schematic representation of T-DNA of binary plasmids. pCAMBIA2301.

**Supplementary Figure 2:** Southern blot analysis of genomic DNA of transgenic lines digested with *Hind*III restriction enzyme. Lanes (1-14) transgenic lines, NT- non-transgenic control plant, M- DIG-labeled molecular weight marker, P- plasmid DNA digested with *Hind*III.
