# Supplementary figures and images for: A High-Throughput Regeneration and Transformation Platform for Production of Genetically Modified Banana
Source: Front Plant Sci. 2015 Nov 26;6:1025. doi: 10.3389/fpls.2015.01025 (PMC4659906; doi:10.3389/fpls.2015.01025)

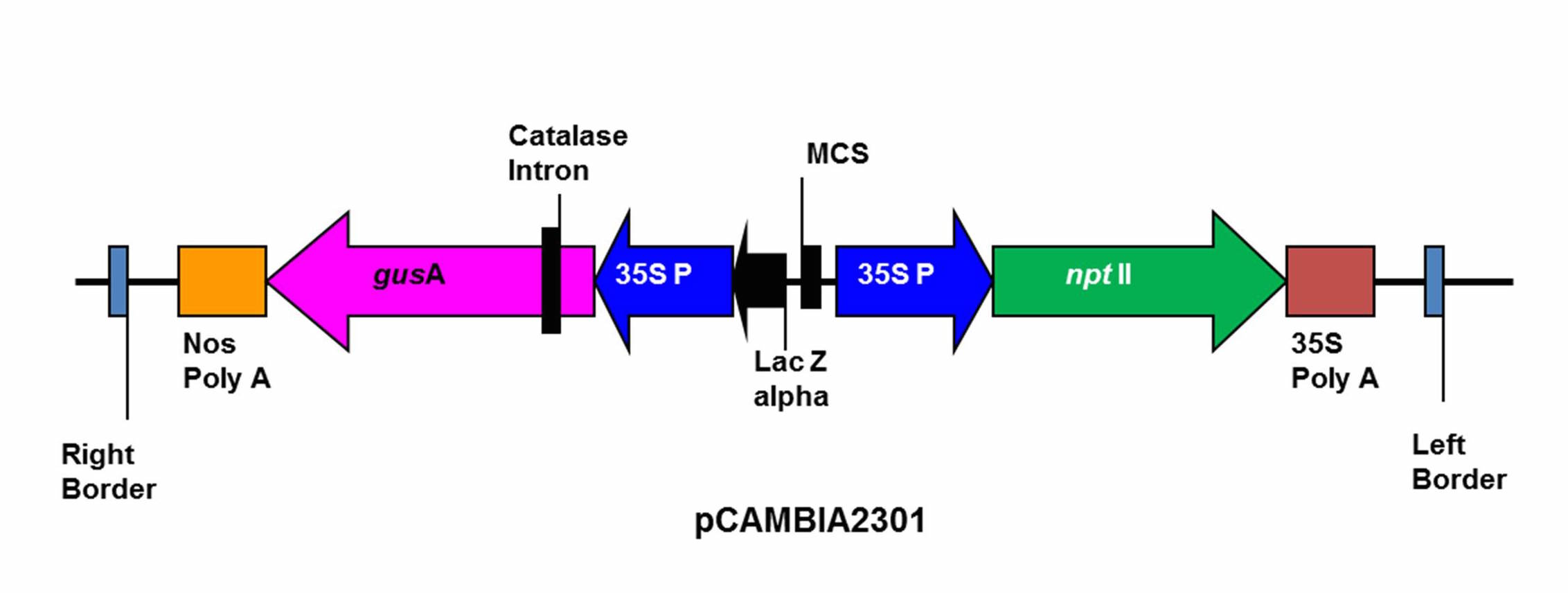

Supplement: Supplementary file 2 [file Image1.JPEG]

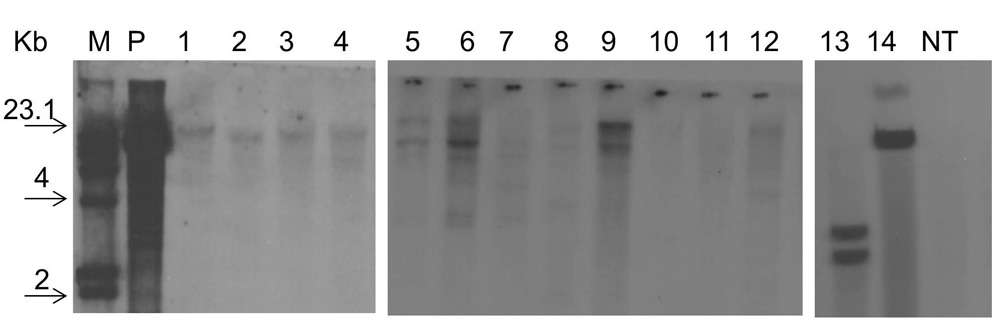

Supplement: Supplementary file 3 [file Image2.JPEG]
